# The value of avian genomics to the conservation of wildlife

**DOI:** 10.1186/1471-2164-10-S2-S10

**Published:** 2009-07-14

**Authors:** Michael N Romanov, Elaina M Tuttle, Marlys L Houck, William S Modi, Leona G Chemnick, Marisa L Korody, Emily M Stremel Mork, Christie A Otten, Tanya Renner, Kenneth C Jones, Sugandha Dandekar, Jeanette C Papp, Yang Da, NISC Comparative Sequencing Program, Eric D Green, Vincent Magrini, Matthew T Hickenbotham, Jarret Glasscock, Sean McGrath, Elaine R Mardis, Oliver A Ryder

**Affiliations:** 1 Genetics Division, San Diego Zoo's Institute for Conservation Research, Zoological Society of San Diego, Arnold and Mabel Beckman Center for Conservation Research, 15600 San Pasqual Valley Rd., Escondido, CA 92027, USA; 2 Dept. of Biology, Indiana State University, Science 285E, 403-25 North 6th St., Terre Haute, IN 47809, USA; 3 Genetic Identification Services, 9552 Topanga Canyon Blvd., Chatsworth, CA 91311, USA; 4 UCLA Sequencing and Genotyping Core, Dept. of Human Genetics, 5309 Gonda, 695 Charles E. Young Dr. South, David Geffen School of Medicine at UCLA, Los Angeles, CA 90095, USA; 5 Dept. of Animal Science, 265D Haecker Hall, University of Minnesota, 1364 Eckles Ave., St. Paul, MN 55108, USA; 6 Genome Technology Branch and NIH Intramural Sequencing Center (NISC), National Human Genome Research Institute, National Institutes of Health, 50 South Dr., Bldg. 50, Rm. 5222, Bethesda, MD 20892, USA; 7 Washington University Genome Sequencing Center, Washington University School of Medicine, Campus Box 8501, 4444 Forest Park Ave., St. Louis, MO 63108, USA; 8 BACPAC Resources, Children's Hospital Oakland Research Institute, 747 52nd St., Oakland, CA 94609, USA

## Abstract

**Background:**

Genomic studies in non-domestic avian models, such as the California condor and white-throated sparrow, can lead to more comprehensive conservation plans and provide clues for understanding mechanisms affecting genetic variation, adaptation and evolution.

Developing genomic tools and resources including genomic libraries and a genetic map of the California condor is a prerequisite for identification of candidate loci for a heritable embryonic lethal condition. The white-throated sparrow exhibits a stable genetic polymorphism (i.e. chromosomal rearrangements) associated with variation in morphology, physiology, and behavior (e.g., aggression, social behavior, sexual behavior, parental care).

In this paper we outline the utility of these species as well as report on recent advances in the study of their genomes.

**Results:**

Genotyping of the condor resource population at 17 microsatellite loci provided a better assessment of the current population's genetic variation. Specific New World vulture repeats were found in the condor genome. Using condor BAC library and clones, chicken-condor comparative maps were generated. A condor fibroblast cell line transcriptome was characterized using the 454 sequencing technology.

Our karyotypic analyses of the sparrow in combination with other studies indicate that the rearrangements in both chromosomes 2^m ^and 3^a ^are complex and likely involve multiple inversions, interchromosomal linkage, and pleiotropy. At least a portion of the rearrangement in chromosome 2^m ^existed in the common ancestor of the four North American species of *Zonotrichia*, but not in the one South American species, and that the 2^m ^form, originally thought to be the derived condition, might actually be the ancestral one.

**Conclusion:**

Mining and characterization of candidate loci in the California condor using molecular genetic and genomic techniques as well as linkage and comparative genomic mapping will eventually enable the identification of carriers of the chondrodystrophy allele, resulting in improved genetic management of this disease.

In the white-throated sparrow, genomic studies, combined with ecological data, will help elucidate the basis of genic selection in a natural population. Morphs of the sparrow provide us with a unique opportunity to study intraspecific genomic differences, which have resulted from two separate yet linked evolutionary trajectories. Such results can transform our understanding of evolutionary and conservation biology.

## Background

### Introduction

Genomic studies in a variety of mammalian taxa have contributed to the development of more comprehensive plans for their conservation, as well as to our understanding of the generation and maintenance of genetic diversity in general (e.g., [1-3]). With the advances of genomic resources for other species, it is now feasible to expand investigation to additional taxonomic realms.

Non-domesticated species of birds are emerging as new animal models with great potential to advance comparative avian genomics and contribute to conservation efforts for threatened species. Birds show incredible diversity in morphology, physiology, and behavior – all of which are analogous to phenotypic variation encountered in other species. With the completion of the chicken genome, as well as significant advances in turkey and zebra finch genomic resources, it is now possible to examine the genetic bases of complex traits using "tools" borrowed from these species. There is a special need for genomic studies in endangered birds to secure their recovery in natural habitats and to increase their resistance to potential threats (e.g., disease, anthropogenic effects). In addition, because many avian species are relatively easy to observe in the wild, they have been intensely studied for decades and, as a result, several long-term studies of their behavior, ecology, and evolution now exist (e.g., the pied flycatcher, *Ficedula hypoleuca*, the great tit, *Parus major*, and the blue tit, *Parus caeruleus*; see for review [4,5]), making it possible to examine adaptive genetic variation. Finally, many avian species are the proverbial "canary in the coal mine", providing us with indicators of the health of an ecosystem and sentinels for the impacts of environmental changes.

In this paper, we review the recent genomics advances in two model avian species, the California condor (*Gymnogyps californianus*) and the white-throated sparrow (*Zonotrichia albicollis*). The critically endangered condor has been affected by habitat destruction and an extreme bottleneck. The white-throated sparrow is an indicator species for the Northern Boreal forest, an essential habitat for avian biodiversity and a key carbon sink [6]. Through studies of their genomes, we will develop a deeper understanding of the genetic mechanisms affecting variation, adaptation, evolution, and behavior in these species, thereby obtaining new information relevant to conservation management of wild populations.

### California condor

The management of wild avian species, including those that are critically threatened and/or endangered, has greatly benefited from the recent advances in genetics and genomics. Among the most striking examples of genetic application to conservation of an endangered species is the California condor (*Gymnogyps californianus*). The condor is an avian icon of California that belongs to the New World vulture family (*Cathartidae*). Over the last two centuries, there has been a rapid decline in the condor population as their natural habitat dramatically shrank. In the 1950s, wild condors were thought to be restricted to Los Padres National Forest in central California. By the early 1980's, a California condor rescue and restoration program was set in motion. Captive propagation was quite successful, and condors have been reintroduced to central and northern California (including Los Padres National Forest and Monterey County), the Grand Canyon area in Arizona, and Baja California del Norte, Mexico [7].

**Figure 1:**
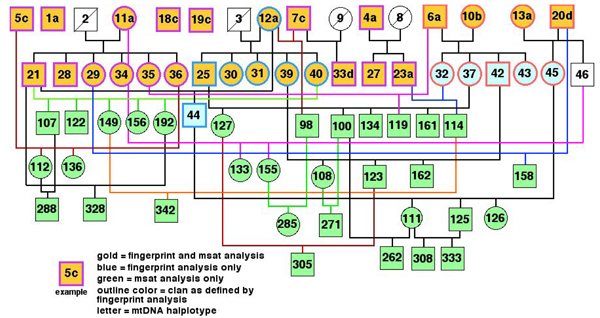
**California condor pedigree of 69 individuals used for genetic studies**. A pedigree includes founders and early generation offspring used for the DNA fingerprint [8], mtDNA [9] and microsatellite (msat; current study) analyses. All individuals are designated using their studbook numbers.

Captive breeding necessitated the initiation of genetic studies to assess the diversity of the remaining population. Early genetic studies of the condor were done at the San Diego Zoo's Institute for Conservation Research, and included the genotyping of the original population with RFLPs [8] and mtDNA [9] analyses (Figure 1). Within the founder population, three unrelated groups (clans) of closely related individuals were identified based on the analysis of multi-locus RFLP fingerprinting [8]. Using mtDNA markers, four distinct maternal lineages were then identified [9]. Finally, karyotype and molecular techniques were developed for sexing condors, involving first an RFLP approach and later utilizing conserved avian sex chromosome sequences for PCR primers [9]. Together, these findings were used to recreate the hypothetical founding generation of California condors, an essential step in developing an effective population management strategy [10].

Genetically small populations are at increased risk of impacts of deleterious mutations as a result of founder effect and genetic drift. A lethal mutation was identified in the expanding pedigree of California condors that negatively affected limb development [11]. The mutant phenotype, called chondrodystrophy, is similar to genetic disorders observed in the chicken (nanomelia) [12], turkey [13], Japanese quail [14], mouse [15,16], and human [17]. Ralls et al. [11] found that the embryonic lethal condition of chondrodystrophy segregates in the affected pedigree as a Mendelian character in a manner consistent with an autosomal recessive mode of inheritance.

With the advent of the genomic era, a detailed comparative molecular cytogenetic analysis of condor chromosomes (GCA) was carried out using chicken macrochromosome (GGA1-GGA9, GGAZ, and GGAW) paints, revealing that the condor has 80 chromosomes [18], comparable to the chicken and turkey karyotypes. This study showed a great homology of chicken and condor macrochromosomes, except for GGA4 that corresponds to GCA4 and GCA9, and suggested an incomplete differentiation of the condor sex chromosomes in the evolution [18].

These studies laid the groundwork for application of genomic technologies in support of California condor conservation efforts. Construction of a genetic map of the California condor and identification of linked markers for the chondrodystrophy mutation were primary goals. The availability of the chicken genome sequence [19] and a large-insert BAC library that was generated at the BACPAC Resources Center, Children's Hospital Oakland Research Institute (CHORI) [20] were crucial components of these efforts. The BAC library consists of 89,665 clones and provides approximately 14-fold coverage of the condor genome. Based upon 172 probes, mostly available from the chicken genome project, we screened the library and produced a first-generation BAC-based chicken-condor comparative map that contained 93 loci.

Among the genes identified in the condor BAC library, several are candidates for chondrodystrophy because they are involved in bone and tissue formation. One such candidate is aggrecan (*ACAN*), which is an integral part of the extracellular matrix [21]. The aggrecan molecule consists of three globular domains, two chondroitin sulfate domains, and one keratin region. Aggrecan molecules attach to a long hyaluronan molecule and form aggregates. Moreover, this functional candidate gene was found to cause skeletal dysplasia in model organisms [12,13,15-17]. We detected two BAC clones positive for the condor *ACAN *gene, one of which was sequenced. As expected, the condor gene sequence showed a great similarity to the chicken homolog [20]. Building on these data, we have continued to investigate the genetic basis of disease in this species. The development and application of practical approaches for management of deleterious genes identified in small populations, including diagnosis of risk factors, identification of unaffected carriers, and breeding strategies to retain genetic variation in such populations with segregating lethal mutations will find precedent in these initial studies.

### White-throated sparrow

The white-throated sparrow (*Zonotrichia albicollis*; Family *Emberizidae*) is a socially monogamous passerine that breeds almost exclusively (>85% of the breeding population) in the North American boreal forest. With large declines in species abundances, increasing evidence for global warming and acid rain, and with the realization that the amount of boreal forest currently protected will not sustain migratory bird populations [6], conservation efforts have begun to focus on such key areas before it is too late. An essential component of a valid conservation plan will include the study of the genomes of boreal species, such as the white-throated sparrow, for the amount and kind of adaptive genetic variation they actually possess, and its distribution across varying habitats.

**Figure 2:**
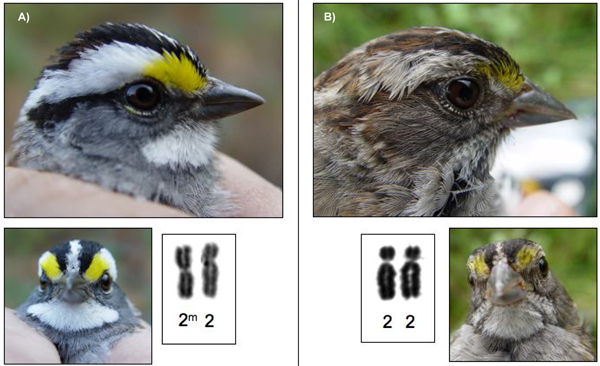
**Morphs of the white-throated sparrow**. Photographs of the morphs of the white-throated sparrow; A) white morphs are heterozygous for a chromosomal rearrangement on autosome 2, while B) tan morphs are homozygous for the non-inverted form of autosome 2.

The white-throated sparrow has several important characteristics that make it an ideal model for the study of conservation genomics. The species is polymorphic, and both sexes occur as either tan (T) or white (W) morphs based on the color of their crown stripes (Figure 2). In addition to morphology, behavioral and life-history characteristics differ between the morphs as well [22-25] (Table 1). In general, white birds are more aggressive than tan birds, yet white are not dominant to tan. Males of the two morphs employ alternative reproductive strategies based on the tradeoffs between current and future reproduction. White males invest heavily in securing matings through song, territorial intrusion, bigamy, and the pursuit of promiscuity (i.e. extra-pair copulations) at the expense of mate-guarding and paternal care [22,23,26-28]. White males have higher levels of circulating testosterone than tan males early in the breeding season [29], most likely because they tend to establish territories in areas of high density where encounters with conspecifics are frequent [24]. By contrast, tan males invest in monogamy and high levels of parental care, and tend to establish territories in more isolated areas. White and tan females seem to exhibit similar trade-offs between investment in parental effort and investment in reproductive effort.

**Table 1:** Characteristics of morph/sex classes in the white-throated sparrow. Characteristics of morph/sex classes; all data from [22-24] except where noted: (1), [76-80]; (2), [26-28,81]; (3), [22,82]. "?" indicates where current knowledge is lacking.

Male comparison			Behavioral traits	Female comparison		
White males	>	Tan males	song rate	White females	>	Tan females
						
	>		aggression (1)		>	
						
	>		intrusion behavior		**?**	
						
	<		mate-guarding		N/A	
						
	N/A		solicitation rate		>	
						
	>		pursuit of promiscuity		**?**	
						
	>		risk of extra-pair paternity		<	
						
	<		risk of brood parasitism		>	
						
	<		parental care (2)		<	
						
	<		mate preference (3)		>	

Interestingly, morph was absolutely correlated with a chromosomal polymorphism resulting from what was believed to be a pericentric inversion of the 2^nd ^largest autosome [30,31]. White birds are heterozygous for the rearrangement (i.e. 2^m^/2, where 2^m ^represents the inverted chromosome 2), and tan birds are homozygous non-carriers (i.e. 2/2). The autosomal inversion segregates in a Mendelian fashion [31]. White-throated sparrows mate disassortatively with respect to the polymorphism [22,23,31], which maintains both morphs in the population [31] and results in parental "phenotypes" that differ in the amount and type of care provided. These parental types respond differently to environmental conditions (e.g., predation, disease, and long-term climatic factors) by altering the condition, sex, and morph of their own offspring through mechanisms that persist across generations.

Results gathered from detailed cytogenetic and molecular analyses in the white-throated sparrow can be combined with long-term data on behavior, physiology, and ecology to reveal the bases of adaptive variation in a natural population. Morphs of the sparrow are somewhat analogous to natural "inbred" lines, where selective factors affecting fitness can be linked to specific genes. In this species, we have the unique opportunity to transcend from genotype to ecosystem. Such results can transform our understanding of evolutionary and conservation biology. In addition, results can be generalized to endangered and at-risk species, providing conservation biologists with a strong foundation with which to build their captive and reintroduction plans.

## Methods

### DNA samples

A condor female named Molloko, studbook (SB) #45, the first ever condor chick hatched in captivity, was selected as the DNA source for constructing the condor microsatellite and BAC libraries. Sequences of microsatellite and BAC clones from Molloko have been deposited in the GenBank and Trace Archive.

For genetic diversity and linkage studies, a set of 121 condor DNA samples including that of Molloko was chosen. These samples were previously isolated for fingerprinting studies and/or gender determination and represent a pedigree of related individuals from four generations and seven families (Additional file 1, created using a computer program Pedigraph, Version 2.4  [32]). This pedigree serves as a condor resource population for further microsatellite and linkage analyses, and also involves three chondrodystrophic individuals, SB #60 and eggs #1405 and #2537.

White-throated sparrow blood samples were collected from individuals that were either part of the long-term study population at the Cranberry Lake Biological Station, New York, or from a captive population housed in research aviaries at Indiana State University. Additional blood samples were also collected from junco *(Junco hyemalis hyemalis*), white-crowned sparrow (*Zonotrichia leucophrys*), song sparrow (*Melospiza melodia*), swamp sparrow (*M. georgiana*), and purple finch (*Carpodacus purpureus*). All blood was separated via centrifugation, and the red blood cells were stored in a lysis buffer at 4°C until needed for further analyses.

Tissue samples from the golden-crowned sparrow (*Z. atricapilla*), Harris's sparrow (*Z. querula*), as well as the majority of white-crowned sparrows *(Z. leucophrys*) and juncos (*J. h. oreganus*, *J. h. caniceps*), were provided by the Museum of Vertebrate Zoology (University of California, Berkeley) and the University of Alaska Museum of the North. Finally, blood samples from the rufous-collared sparrow (*Z. capensis*) were kindly provided by Ignacio T. Moore, Department of Biological Sciences, Virginia Polytechnic Institute and State University.

### Microsatellite-based analyses

A California condor microsatellite-enriched library was created at Genetic Identification Services, following the company's protocol, and includes eight sublibraries, A to H, with enrichments for repeat motifs CA, ATG, TACA, TAGA, AAG, AAT, AAAT, and CATC, respectively. For marker design, approximately 180 random clones were sequenced. Dye-labeled primers for a set of 17 informative microsatellite markers identified by Genetic Identification Services (Table 2) were used to amplify condor DNA. These included 13 tetra-, two tri- and two dinucleotide repeat loci. Three more avian microsatellite markers, *FhU2*, *HrU2*, and *HrU6 *(Accession Numbers X84361, X84087, and X84091) [33,34], were tested using Schuelke's [35] procedure for the fluorescent labeling of PCR fragments with the M13(-21) universal primer.

**Table 2:** Summary of genetic population statistics for 17 condor microsatellite loci.

Locus*	*na*	*ne*	*I*	*Ho*	*He*	***F***_*IS*_	*PIC*	Test for HWE	
								
								Chi-square	P
*A*8	5	3.025	1.2865	0.8091	0.6725	-0.2086	0.6150	35.6162	0.0001
*A*20	2	1.9468	0.6794	0.5868	0.4884	-0.2065	0.3681	4.9577	0.0260
*B*7	2	1.3006	0.3927	0.2500	0.2321	-0.0817	0.2044	0.7361	0.3909
*C*5 (EF108178)	3	2.0958	0.7928	0.7179	0.5251	-0.3731	0.4104	20.5154	0.0001
*D*6	3	2.1513	0.8749	0.5470	0.5375	-0.0221	0.4526	1.4111	0.7029
*D*9 (EF108179)	3	2.4596	0.9961	0.7500	0.5959	-0.2638	0.5274	15.3954	0.0015
*D*10	3	2.152	0.8771	0.5500	0.5376	-0.0274	0.4537	1.2557	0.7397
*D*24	2	1.9692	0.6853	0.4583	0.4942	0.0688	0.3711	0.6391	0.4240
*D*126	2	1.3318	0.4154	0.1583	0.2502	0.3645	0.2181	16.5900	0.0000
*G*8	2	1.9687	0.6852	0.3529	0.4941	0.2827	0.3710	9.8007	0.0017
*H*3	2	1.894	0.6649	0.3118	0.4746	0.3394	0.3606	11.0699	0.0009
*H*6	2	1.9799	0.6881	0.6134	0.4970	-0.2395	0.3724	6.5885	0.0103
*H*106	2	1.9760	0.6871	0.5679	0.4960	-0.1495	0.3719	2.4915	0.1145
*H*115 (EF108180)	2	1.2236	0.3288	0.2034	0.1835	-0.1132	0.1660	1.4438	0.2295
*H*127	2	1.1918	0.2984	0.1765	0.1616	-0.0968	0.1480	1.0573	0.3038
*H*238	2	1.3034	0.3948	0.2521	0.2337	-0.0831	0.2057	0.7558	0.3846
*H*269 (EF108181)	2	1.4449	0.4863	0.3306	0.3092	-0.0736	0.2605	0.5906	0.4422

Abbreviations: *na*, observed number of alleles; *ne*, effective number of alleles; *I*, Shannon's information index; *Ho*, observed heterozygosity; *H*, gene diversity ; *F*_*IS*_, Wright's  fixation index (as a measure of heterozygote deficiency or excess); *PIC*, polymorphism information content; HWE, Hardy-Weinberg equilibrium (deviations are given in bold); *P*, probability.

* GenBank Accession Number is given in the parentheses, if available.

**Figure 3:**
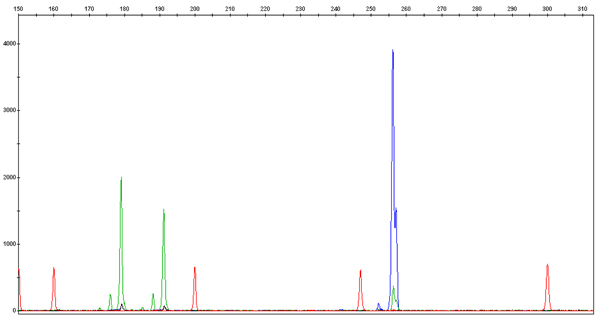
**An example of microsatellite genotyping profile at the loci *C5 *and *D6***. The DNA from a condor male (SB #162) was amplified with the *C5 *and *D5 *primers labeled with two different fluorescent dyes. The bird is heterozygous for *C5 *(green alleles, 179 and 191 bp) and homozygous for *D5 *(blue alleles, 256 bp both). Red peaks are size standards. Values along the y-axis represent the intensity of fluorescence (a.u.), values along the x-axis mark the size of the DNA fragments (a function of time).

The PCR products were run on an ABI 3100, ABI 3130 or ABI 3730 Genetic Analyzer instruments (Applied Biosystems), the fragments or alleles generated were verified (Figure 3), and the data analyzed for population genetic parameters and linkage relationships. The following computer programs were used for estimating population genetic statistics: Microsatellite Tools for Excel, Version 3.1 [36], and POPGENE, Version 1.31 [37]. A preliminary linkage analysis was done using Locusmap, Version 1.1 [38].

Additionally, over 1,900 random clones from the same microsatellite-enriched library were bi-directionally sequenced at High-Throughput Sequencing Solutions (University of Washington, Seattle). These were then used to develop additional polymorphic microsatellite markers using the Primer3 online program [39] for primer design. All sequences have been deposited in the GenBank (Accession Numbers DQ471953, DQ483109 – DQ484036, EF108178 – EF108181, EF116886 – EF116903) and analyzed for the presence of repetitive elements using a package of NCBI BLAST programs [40] and RepeatMasker http://www.repeatmasker.org/[41].

### BAC library screen

A second library screening was carried out using the CHORI protocol http://bacpac.chori.org/overgohyb.htm and a four-dimensional filter hybridization procedure [42], with modifications [20]. The latter, in brief, included the simultaneous ^32^P-labeling of 216 overgo probes (Additional file 2) and their hybridization to BAC filters, with variable 36 overgo pools per filter per dimension. This strategy allowed the completion of a total of 24 hybridizations in a relatively short amount of time. The overgo probes were available from the previous chicken and zebra finch genome projects [43,44] or newly designed on the basis of available condor and chicken sequences (Additional file 2). Other screening procedure details and overgo sequences can be provided by the authors upon request.

Thirteen condor BAC clones were subsequently sequenced at NIH Intramural Sequencing Center (NISC) following protocols described in [45], and their sequences are publicly available from GenBank (Accession Numbers AC171379, AC171743, AC172166, AC183448 – AC183451, AC183845, AC183846, AC188502 – AC188504, and AC191192).

### Cytogenetic techniques

Avian fibroblast cell lines from feather pulp were established at the San Diego Zoo's Institute for Conservation Research as described in [46]; these cell lines were used for karyotyping and fluorescence *in situ *hybridization (FISH).

Metaphase chromosomes were obtained by arresting cell division with colcemid (Gibco BRL), hypotonic treatment with 0.075 M KCl, and fixation in 3:1 methanol:acetic acid. To assess chromosome number, we stained chromosome preparations with 5% Giemsa (Sigma) in 0.07 M phosphate buffer (pH 6.8). To examine banding patterns, some slide preparations were trypsin G-banded following [47].

BAC DNA was used as FISH probes and extracted following standard procedures. After extraction from a BAC clone, DNA was purified and labeled with a fluorochrome according to the manufacturer's guidelines (Vysis, Abbott Laboratories).

### Fragment and sequence analysis

Fragments from coding regions of the *ACAN *candidate gene were PCR amplified and sequenced using an ABI 3100 or ABI 3130 Genetic Analyzer. Sequencher (Gene Codes) was employed for post-sequencing analysis.

A condor fibroblast cell line was utilized for mRNA extraction and consequent generation of cDNA sequences at Washington University Genome Sequencing Center using the 454 sequencing technology [48]. The total RNA was used to prepare a polyT-primed cDNA library by an in-house version of the Clontech SMART protocol without normalization [49]. The 454 sequence data set has been deposited in Trace Archive.

**Figure 4:**
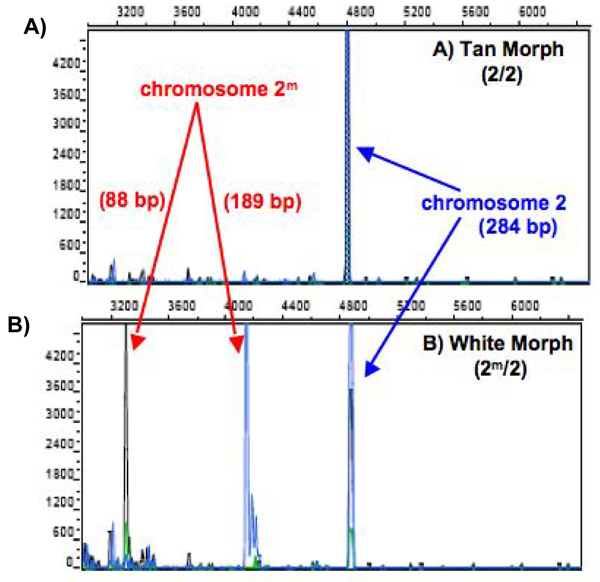
**Molecular assay for morph determination**. Molecular assay for morph determination in the white-throated sparrow, modified in the Tuttle laboratory for use on automated sequencers from primers described in [50]. Panel A shows a tan morph with a single peak at 284 bp amplified from chromosome 2. Panel B shows a white morph with a band for chromosome 2 (at 284 bp) and two additional bands at 88 bp and 189 bp, which occur when PCR product amplified from chromosome 2^m ^is digested with restriction endonuclease *Dra*I. If they occur in nature, we assume that birds homozygous for 2^m ^("super whites") would produce a pattern showing only the 88 bp and 189 bp bands. This technique has been verified in adults of known plumage. Values along the y-axis represent the intensity of fluorescence (a.u.), values along the x-axis mark the size of the DNA fragments (a function of time).

For analyses of the white-throated sparrow morph, we modified the Michopoulos et al. [50] protocol to amplify a portion of the vasoactive intestinal peptide (*VIP*) gene located on chromosome 2 that either has a *Dra*I restriction site (the "2^m^" state) or lacks it (the "2" state). Our modifications included fluorescently labeling both the forward and reverse primers so that all products could be visualized on an ABI PRISM 310 Genetic Analyzer, and reducing PCR reactions to a final volume of 10 μl. Figure 4 shows the genetic analyzer output for white and tan morphs. To examine evolutionary history, we used this same "morph" assay on DNA samples collected from several related passerine species.

## Results

### California condor studies

#### Genetic diversity and linkage analysis

For genetic diversity assessment, linkage analysis and construction of the condor genetic map, we chose 121 related individuals that formed a condor resource population. Their genotyping at 17 microsatellite loci resulted in the estimates of within-population genetic variation shown in the Table 2. The average number of alleles was 2.41 ± 0.80 per locus, the effective number of alleles 1.85 ± 0.50, and the Shannon's information index 0.66 ± 0.26. The average heterozygosity for all loci was 0.45 ± 0.01, and the genetic diversity 0.42 ± 0.04. For eight of the 17 loci, there were deviations from Hardy-Weinberg equilibrium.

A preliminary linkage analysis suggested that among the 17 microsatellite loci we may expect a linkage between loci *D10 *and *D6 *(LOD score = 21.67), as well as between *A20 *and *D9 *(LOD score = 5.12) and between *B7 *and *H238 *(LOD score = 5.12).

To expand the microsatellite mapping subproject, we tested three more avian microsatellite markers, *FhU2*, *HrU2*, and *HrU6 *[34,35] that previously showed satisfactory amplification results in a wide range of other birds, including some New World vultures. We were able to amplify the condor *FhU2 *and *HrU2 *PCR fragments, while *HrU6 *did not work on condor DNA in our hands.

Additionally, we obtained over 900 clone sequences from the microsatellite-enriched library and derived from them around 300 new markers for genotyping the resource population at the next project stage.

#### *In silico *mapping

In total, we obtained approximately 1000 short genomic sequences from the microsatellite-enriched library. In the course of *in silico *mapping, we found that 30% of these are homologous to chicken sequences across almost all chicken chromosomes (GGA1-GGA15, GGA17, GGA19-GGA21, GGA23, GGA24, GGA26-GGA28, GGAZ, and UN) and some other avian sequences. Many of these sequences contain microsatellites and other repetitive elements (LINEs, retroviral LTRs, and satellites) known in chicken. We also found satellite sequences previously detected by Keyser et al. [51] that only occur in New World vultures.

#### BAC library screening

The second screen of a 2.8× subset of the condor BAC library was done using 216 overgo probes. The latter were mostly available from the zebra finch and chicken genome projects [20] or derived from the condor microsatellite library clone sequences (Additional file 2). As a result, we added 100 more loci onto the BAC-based chicken-condor comparative map, and it currently contains 199 loci (Additional file 3). At present, the total number of condor BAC-gene or other sequence assignments is almost 670 (Additional file 4).

#### FISH mapping

**Figure 5:**
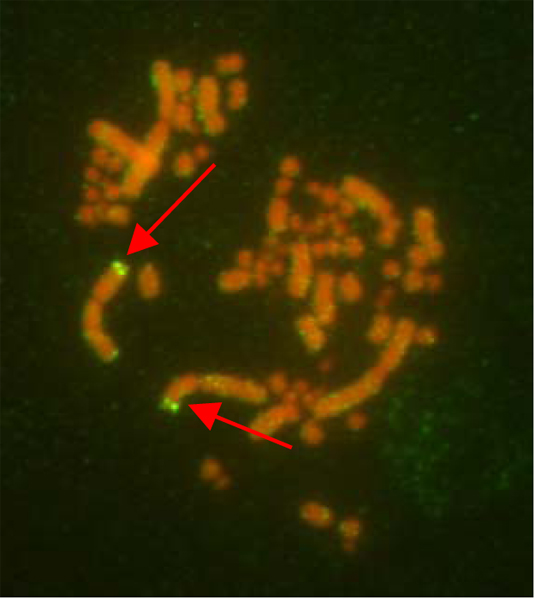
**California condor FISH mapping**. An example of FISH using metaphase chromosomes of the California condor and a biotinylated BAC clone containing the *ACVR2B *gene located on GCA2.

To verify the efficiency of cross-species hybridization of the condor BAC library and to build the condor cytogenetic map, we performed FISH using more than 50 condor BAC clones that were positive for targeted condor genes (Figure 5). Most BACs were found to be orthologous to the appropriate chicken genes and chromosomes (Additional file 5). We identified one intrachromosomal rearrangement on chromosome 4 (the *FGF2 *locus), with more intrachromosomal rearrangements on the Z chromosome.

#### Candidate gene hunting

Among the positive clones for almost 200 genes identified in the condor BAC library, we selected and sequenced one that harbors the *ACAN *gene (CH262-21P20, Acc. No. AC171743). Based on the sequence information, we designed PCR primers to re-sequence the entire gene in affected chicks, their parents, as well as unaffected non-relatives. After sequencing coding regions of the gene, we have yet to detect SNPs that could be associated with the chondrodystrophy.

#### Comparative human-condor mapping

To date, NISC has generated sequence of 13 condor BACs, each 100–200 kb in size. These sequences are orthologous to human chromosome 7 and six chicken chromosomes. Based on the sequences, a condor-human comparative physical map for a region on HSA7 was designed and is available online through the NISC web site (Additional file 6). After comparing against the chicken whole genome sequence using BLAST, the 13 sequenced BACs were also added onto the BAC-based chicken-condor comparative map (Additional file 3).

#### 454 sequencing

**Figure 6:**
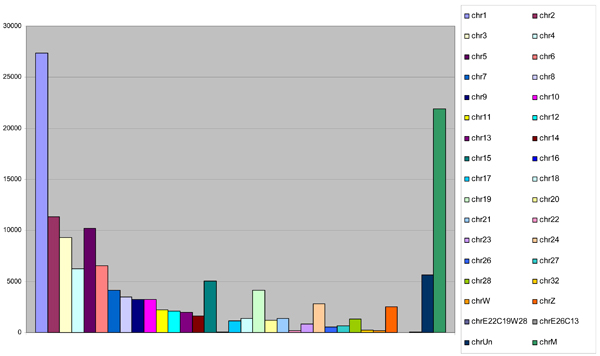
**Distribution of the condor 454 transcripts across the chicken chromosomes**. The distribution of the condor transcripts based on their orthology to the chicken chromosomes.

At the Washington University Genome Sequencing Center, almost 440,000 cDNA sequences were generated from a fibroblast cell line using the 454 technology. The total length of these short transcripts (about 100 bp each) is 43,379,069 bp. We found that 78% transcripts were homologous to chicken genes, while being distributed across almost all chicken chromosomes (Figure 6). After assembly and clustering, we were left with around 15,000 contigs that contained about 190,000 reads.

Surprisingly, for a number of genes expressed in this cell line, we had unusually high numbers of reads, with the most extreme transcript having over 55,000 reads (Additional file 7). Amongst the most abundant transcripts (Additional file 8), there were several proteins that are expected to be expressed in fibroblast cell lines, like proteoglycans, a keratin associated protein, fibroblast growth factor receptor 1 and alpha-actin, and other housekeeping proteins. However, one protein, the mitotic arrest deficient-like 1 (MAD1L1), had especially higher levels of expression in this cell line.

### White-throated sparrow studies

#### Chromosomal studies

**Figure 7:**
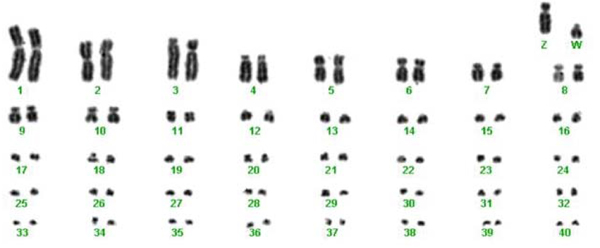
**Complete karyotype of white female**. Complete karyotype of white (2^m^/2) female (Z/W) KB15793, showing 82 macro- and microchromosomes, including 40 autosome pairs and two sex chromosomes (Z and W); data generated at the San Diego Zoo's Institute for Conservation Research, Zoological Society of San Diego. In addition to the rearrangement of 2, chromosome 3 also sometimes exhibits an alternate form (3^a^; as seen here).

**Figure 8:**
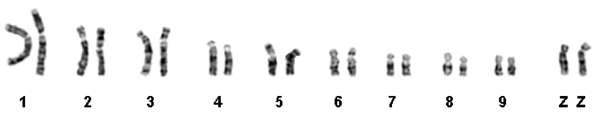
**G-banded chromosomes 1–9 and Z from the sparrow**. G-banded chromosomes 1–9 and Z from a 2^m^/2 3^a^/3 white-throated sparrow (KB15794; San Diego Zoo's Institute for Conservation Research, Zoological Society of San Diego).

To confirm previous studies and as a necessary first step in genomic studies, we established eight white-throated sparrow cell lines (six white, and two tan; KB15792 – KB15799) accessioned in the Frozen Zoo^® ^at the Zoological Society of San Diego. Using standard Giemsa staining, we confirmed that there is a total diploid chromosome number of 82, including the Z and W sex chromosomes (Figure 7). G-banding comparisons indicate that the rearrangements distinguishing 2^m ^from 2 and 3^a ^from 3 are complex. The banding differences in each pair involve the centromere and cannot be resolved by inverting only one segment of the chromosome and thus are likely the result of more than one inversion on each chromosome (Figure 8).

Karyotypic analyses of 46 randomly selected white-throated sparrows (Table 3) indicate that with regards to chromosome 2 and 3, the most common genotypes are 2/2 3^a^/3^a ^(tan; type C) and 2^m^/2 3^a^/3^a ^(white; type F). Homozygous genotypes 2^m^/2^m ^and 3/3 are rare [31] or nonexistent (this study). Most of the chicks from tan male – white female pairs (T × W) were the type F genotype (2^m^/2 3^a^/3^a^); few of their chicks (approximately 15%) were heterozygous for chromosome 3 (3^a^/3). By contrast, offspring from white male – tan female pairs (W × T) were fairly evenly split between types B (2/2 3^a^/3), C (2/2 3^a^/3^a^), and F (2^m^/2 3^a^/3^a^); and, over 33% of their chicks were 3^a^/3 heterozygotes.

**Table 3:** The percentage of white-throated sparrows sampled having various arrangements of chromosomes 2 and 3. Table adapted from [31]. Types A-C are all tan, whereas types D-G are all white.

Studies	Tan			White			
	Type A 2233	Type B **2233**^a^	Type C **223**^a^**3**^a^	Type D **22**^m^**33**	Type E **22**^m^**33**^a^	Type F **22**^m^**3**^a^**3**^a^	Type G **2**^m^**2**^m^**3**^a^**3**^a^
Thorneycroft [31], males (N = 260)	0.45	4.00	32.00	0.45	13.00	50.00	-
This study, males (N = 24)	-	12.50	37.50	-	12.50	37.50	-
Thorneycroft [31], females (N = 137)	1.00	9.00	50.00	-	4.00	34.00	1.00
This study, females (N = 22)	-	9.09	22.70	-	9.09	59.09	-
This study, T × W parents (N = 26)	-	7.7	26.9	-	7.7	57.7	-
This study, W × T parents (N = 12)	-	25.0	33.3	-	8.3	33.3	-

#### Comparative analyses

**Figure 9:**
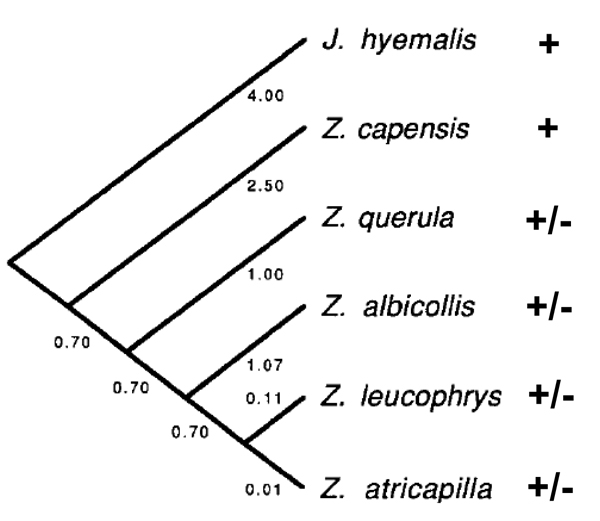
***VIP *restriction site mapped on *Zonotrichia *tree**. Phylogenetic tree for *Zonotrichia *from [75] showing branch lengths (% nucleotide differentiation). Mapped on the tree are presence (+), absence (-), or both presence and absence (+/-) of the *Dra*I restriction site in the *VIP *fragment. Data for the restriction site were derived from Table 1. Only the four North American *Zonotrichia *species show polymorphism for the *VIP *fragment, suggesting that the polymorphism arose in the common ancestor of those four species.

**Figure 10:**
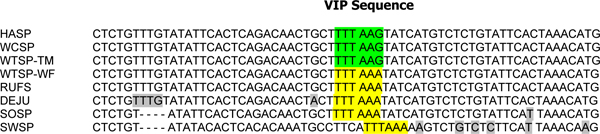
***VIP *sequence for several passerine species**. Sequence alignment for an intron of the vasoactive intestinal peptide (*VIP*) gene (primers described in [50]). Bases highlighted in gray show differences between the white-throated sparrow (*Zonotrichia albicollis*; WTSP of both morphs), Harris's sparrow (*Z. querula*; HASP), the white-crowned sparrow (*Z. leucophrys*, WCSP), the rufous-collared sparrow (*Z. capensis*, RUFS), the dark-eyed junco (*Junco hyemalis*; DEJU), the song sparrow (*Melospiza melodia*; SOSP), and the swamp sparrow (*M. georgiana*; SWSP). Yellow highlighting indicates the *Dra*I restriction site, which is found in dark-eyed juncos and song sparrows, as well as white morphs (W) white-throated sparrows; green highlighting shows the lack of a *Dra*I restriction site found in tan (T) morph white-throated sparrows.

The *VIP *assay for morph is based on the fact that there is a SNP on chromosome 2^m ^that forms a restriction site for *Dra*I, whereas the *VIP *fragment on chromosome 2 does not contain that site [50]. In an assay of 546 white-throated sparrows (Table 4), 50.9% were 2^m^/2 (white) and 49.1% were tan (2/2); no individuals were 2^m^/2^m ^("superwhites"). When we examined this site in the four other species of *Zonotrichia *(*Z. atricapilla*, *Z. querula*, *Z. leucophrys, Z. capensis*), only the North American species of *Zonotrichia *show polymorphism for the *VIP *fragment suggesting that the common ancestor of all four had the polymorphism (Table 4; Figure 9). All 10 rufous-collared sparrows (*Z. capensis*) tested were monomorphic for the *Dra*I restriction site. In addition, other passerine birds tested (three subspecies of junco, including *Junco hyemalis hyemalis*, *J. h. oreganos*, and *J. h. caniceps*, swamp sparrow (*Melospiza georgiana*), song sparrow (*M. melodia*), and purple finch (*Carpodacus purpureus*)) were also monomorphic for the *Dra*I restriction site (Table 4). Preliminary sequence analysis of the *VIP *intron indicates that in the rufous-collared sparrow, the junco, the song sparrow, and the swamp sparrow, the SNP is the same as that found in chromosome 2^m ^of the white-throated sparrow (Figure 10).

**Table 4:** Results of the white-throated sparrow "morph" assay using the *Dra*I restriction site located in the *VIP *gene [50]. Species heterozygous for the *Dra*I restriction site show 3 fragments (F1-F3); species homozygous for the *Dra*I restriction site show 2 bands, one smaller band at approximately 72–89 bp and one larger band at 185–190 bp (F1 and F2); finally, homozygotes that do not carry the *Dra*I restriction site show only a single band at 284 bp (F3).

Species	Subspecies	Genotype	VIP F1 (bp)	VIP F2 (bp)	VIP F3 (bp)	#	%
White-throated sparrow (*Zonotrichia albicollis*)		2^m^/2^m^ Homozygous – *Dra*I site	88	189	-	0	0
		2^m^/2 (White Morph) Heterozygous	88	189	284	278	50.9
		2/2 (Tan Morph) Homozygous – No site	-	-	284	268	49.1

White-crowned sparrow (*Z. leucophyrs*)		Homozygous – *Dra*I site	80	190	-	2	16.7
		Heterozygous	80	190	284	4	33.3
		Homozygous – No site	-	-	284	6	50.0

Golden-crowned sparrow (*Z. atricapilla*)		Homozygous – *Dra*I site	83	190	-	0	0
		Heterozygous	83	190	284	3	100
		Homozygous – No site	-	-	284	0	0

Harris's sparrow (*Z. querula*)		Homozygous – *Dra*I site	72/73	88/91	-	1	25.0
		Heterozygous	72 or 73	88 or 91	284	0	0
		Homozygous – No site	-	-	284	3	75.0

Rufous-collared sparrow (*Z. capensis*)		Homozygous – *Dra*I site	89	191	-	10	100

Dark-eyed junco (*Junco hyemalis*)	Slate-colored *hyemalis*	Homozygous – *Dra*I site	89	189	-	4	100
	Oregon *oreganus*	Homozygous – *Dra*I site	89	189	-	3	100
	Gray-headed *caniceps*	Homozygous – *Dra*I site	89	189	-	1	100

Song sparrow (*Melospiza melodia*)		Homozygous – *Dra*I site	89	185	-	5	100

Swamp sparrow (*M. georgiana*)		Homozygous – *Dra*I site	89	186/189	-	5	100

Purple finch (*Carpodacus purpureus*)		Homozygous – *Dra*I site	89	193	-	1	100

## Discussion

### California condor genetics and genomics

Genetic management of California condors aims to maximize the retention of genetic diversity by minimizing mean kinship within the population. We used a series of mostly tetranucleotide repeat markers to evaluate genetic diversity among individuals obtained from the wild and ascertain linkage relationships between microsatellite loci. The observed level of heterozygosity (0.45) found in the resource population could be considered as a good sign of a successful captive propagation program and population recovery. On the other hand, observation of Hardy-Weinberg equilibrium deviations at eight out of 17 loci might suggest that the current condor population is impacted by inbreeding. These results can be used for validating genetic management recommendations currently used for this endangered species.

Taking advantage of recent progress in genome studies of the domestic chicken, we are developing a genetic map for the California condor. Among the 17 microsatellite loci already used, there are three potential linkages established. Approximately 300 more markers have been designed and, after validation, will be used for genotyping the resource population. This would be the first attempt to make a genetic linkage map for condors, which will be later supplemented with SNP markers to locate and characterize candidate loci for the chondrodystrophy mutation, to identify carriers of the chondrodystrophy allele and to improve genetic management of this and other heritable disease risk-factor loci.

The conventional re-sequencing of *ACAN*, a likely chondrodystrophy candidate gene, was complicated by the compound structure of this gene, including the large number of exons (18 in the chicken) and a complex repetitive structure of its longest exon that encodes for chondroitin sulfate attachment domain. The latter contains in the chicken a variable repeat region that includes 19 repeats, each of 60 nucleotides, with the reported nanomelic stop codon [12]. Although we have not yet been able to identify a causative SNP in coding regions of the condor *ACAN *gene, we continue this investigation in both exonic and intronic sequences as well as other regulatory regions that could also be associated with changes in the *ACAN *regulation. We plan to use a high throughput 454 sequencing technology that may identify or eliminate this locus as the locus of the chondrodystrophy mutation. Construction of the condor linkage map will bring us closer to the mapping of the chondrodystrophy locus and it will enable confirmation of a role for the *ACAN *gene or other (marker) loci linked to the disease. In either scenario, we will be able to develop a molecular assay for heterozygous carriers of the chondrodystrophy mutation.

The 454 cDNA sequence data reported here provides a first insight into the condor transcriptome. Interestingly, *ACAN *was found to be expressed in this cell line. We expect that establishment of fibroblast cell lines for normal and affected chicks will enable direct comparison of full-length cDNA sequences to significantly assist in the identification of the potential chondrodystrophy mutation in *ACAN *or other candidate genes. Such data will provide one more functional genomics approach to pinpoint the disorder mutation.

MAD1L1 was shown to be highly overexpressed in the condor fibroblast cell line analyzed. MAD1L1 is a component of the mitotic spindle-assembly checkpoint, and its mutations are suggested to play a role in the pathogenesis of various types of human cancer (e.g., [52]). Abnormal overexpression of MAL1L1 gene may be linked to a particular phenotype observed in this condor cell line. The cell line has a continuous long-term proliferation and heteroploid features, an indication that the line is transformed possibly due to a mutation affecting MAD1L1 expression. As MAD1L1 is known to be involved in neoplasia, further investigation of this cell line can provide a source of comparative studies and shed light on de-regulation of cell proliferation in avian species.

We were able to obtain dense coverage of the Z chromosome (Additional file 3) with the second BAC library screen. We chose to cytogenetically assign the Z-linked BACs to condor chromosomes using FISH because preliminary indications suggested that there could be intra- and interchromosomal rearrangements in the condor relative to the chicken Z chromosomes. Furthermore, we consider it useful for the future studies to compare Z-linked gene expression in condor males and females to see if there is any difference or if we have a dosage compensation effect similar to what was shown for the zebra finch and chicken [53,54].

Overall, we detected a few changes in gene location and order between chicken and condor chromosomes, indicating a high degree of conserved synteny. Our data support a higher similarity of the cathartid genome to the avian ancestral karyotype and a more basal position of Cathartidae as compared to Accipitridae and Falconidae [55], as well as the general stability of avian genomes over the course of evolution [56]. Another piece of evidence in support of those ideas comes from the fact that the chicken and condor genomes demonstrate a high degree of sequence homology and share many common avian repetitive elements including LINEs, retroviral LTRs, and satellites. However, the presence of satellite sequences specific to New World vultures [51] suggests that some distinctive genomic features have emerged in the cathartids over almost the 100-million years of evolution in this avian lineage. We hypothesize that the specific satellite sequences inherent to the Cathartidae may represent unique genomic signatures that could help resolve the long disputed taxonomic position of this avian family (e.g., [55,57,58]).

The 454 cDNA sequence data, as well as condor microsatellite library clone sequences, have been added to a new avian genomic database *Gallus *GBrowse that includes the whole genome sequence of the chicken as a reference http://birdbase.net/cgi-bin/gbrowse/gallus08/[59]. Along with sequence information for turkey and zebra finch, this database serves as a new powerful tool for avian comparative genomics. All the sequence information available for the condors is also being deposited in GenBank and Trace Archive, adding the condor to the ever-expanding list of avian genomes (Table 5). Finally, there is a web site we have developed for the condor genomics project: https://msu.edu/~romanoff/index2.htm.

**Table 5:** Representation of avian species in the NCBI databases (as of June 8, 2009).

Species/Order	Nucleotide/Protein	Trace records	PubMed
Chicken *(Gallus gallus)/Galliformes*	927,747/35,072	18,186,087	90,868
Zebra finch* (Taeniopygia guttata)/Passeriformes*	288,655 / 17,308	15,499,360	359
Turkey *(Meleagris gallopavo)/Galliformes*	57,934 / 560	77,154	321
Duck *(Anas platyrhynchos)/Anseriformes*	4,888 / 474	---	979
**White-throated sparrow***** (Zonotrichia albicollis)**/**Passeriformes***	**3,955 / 124**	**268,938**	**41**
Domestic pigeon *(Columba livia)/Columbiformes*	2,371 / 244	---	9,669
Red-backed fairy wren *(Malarus melanocephalus)/Passeriformes*	2,026 / 394	---	1
Lazuli bunting *(Passerina amoena)/Passeriformes*	1,791 / 590	---	6
Hwamei *(Garrulax canorus)/Passeriformes*	1,572 / 1,340	---	1
Indigo bunting *(Passerina cyanea)/Passeriformes*	1,572 / 568	---	16
Gadwall *(Anas strepera)/Anseriformes*	1,233 / 514	---	16
California condor (Gymnogyps californianus)/Ciconiiformes*	**976/4**	452,496**	**13**
Yellow wagtail *(Motacilla flava)/Passeriformes*	890/91	---	5
Collared flycatcher (*Ficedula albicollis)/Passeriformes*	770/287	---	56
Adelie penguin *(Pygoscelis adeliae)/Sphenisciformes*	671/17	---	69
European pied flycatcher (*Ficedula hypoleuca)/Passeriformes*	667 / 295	---	86
White-winged fairy-wren *(Malurus leucopterus)/Passeriformes*	639 / 190	---	1
Japanese quail (*Coturnix japonica)/Galliforme*s	634 / 487	---	4,435

*According to [55,57,58] and contrary to NCBI Taxonomy database, the New World vultures are not related to storks (*Ciconiiformes*).

** Includes 419,240 sequences generated with 454 technology and 33,256 entries obtained with shotgun sequencing.

### White-throated sparrow as a behavioral model

Advances in white-throated sparrow genomics have begun at an obvious point – the characterization of the chromosomal rearrangement affecting morphic differences. Thorneycroft [30,31] first karyotyped the species, reporting a diploid chromosome number of 82 or 84, and based on centromere position, presumed pericentric inversions in both chromosome 2 and 3. Here, our karyotypic analyses confirm a diploid number of 82, with the typical avian pattern of approximately 30 pairs of microchromosomes (Figure 7; see [60,61] for reviews of avian microchromosomes). G-banding analyses suggest that the rearrangements in chromosomes 2 and 3 are not simple, but instead consist of multiple inversions involving the centromere. These results are consistent with those reported in Thomas et al. [62], who used comparative cytogenetic mapping to show that 2^m ^and 2 differ by a pair of pericentric inversions spanning at least 98 Mb or greater than 86% of the chromosome. Chromosomal inversions are often adaptive because they can result in co-adapted gene complexes [63], therefore further investigation of gene structure in this species is warranted. However, to be valuable to evolutionary and conservation biology, such studies must be done within the context of the ecological and social environments that selected for the maintenance of polymorphism.

In the white-throated sparrow, recombination is suppressed in heterozygotes for chromosome 2^m^. Thorneycroft [31] found that chiasma did form between the p-arm of chromosome 2 and one arm of chromosome 2^m^, but they did not form between the q-arm of chromosome 2 and chromosome 2 m. In addition, chiasma formed between the p-arm of chromosome 3 and the p-arm of chromosome 3^a^, but not between the q-arms of these chromosomes. Using nine loci within the 2^m ^rearrangement, Thomas et al. [62] confirmed that recombination only occurred in the telomere area between the p-arm of chromosome 2 and chromosome 2^m^, thus restricting gene flow between the two. In addition, they found that chromosome 2 had five times the nucleotide diversity of chromosome 2^m^. Based on these results, Thorneycroft's [31] meiotic data, and our data on population genotype frequencies (Table 3), we predict that chromosome 3^a ^has also evolved under limited recombination and reduced gene flow.

Chromosomal inversions may be disadvantageous if they foster the accumulation of suites of deleterious alleles within the area of low recombination. Since the inversion in the white-throated sparrow is relatively large and covers at least 86% of chromosome 2^m^, it is likely that some negative fitness affects are associated with homozygosity; the same is likely true for chromosome 3^a^. We did not find 2^m^/2^m ^or 3^a^/3^a ^individuals in our karyotypic analyses, nor did we find any 2^m^/2^m ^birds in over 546 individuals sampled in the *VIP *assay. Thorneycroft [31] found that parents with a single 2^m ^chromosome passed this on to half of their offspring however, parents with a single chromosome 3 passed this on to only a quarter of their offspring. Given his results, we would expect that white birds (2^m^/2) pairing with other white birds (2^m^/2) would result in up to 25% lethal or semi-lethal homozygotes. It is surprising then that in our study, the frequencies of particular genotypes differed in the sexes and between the two disassortative pair types (Table 3). Certain combinations of chromosomes 2 and 3 also appeared more viable than others. Together, these factors suggest non-Mendelian transmission, interchromosomal linkage and pleiotropy, and a strong interaction between autosomal genotype and sex. Our laboratory is continuing to investigate the relationship between chromosomes 2 and 3 using a combination of genomics techniques and population data.

Comparative analyses between the white-throated sparrow and its relatives can be quite revealing about genomic evolution. In the rufous-collared sparrow (*Zonotrichia capensis*), Rocha et al. [64] reported polymorphisms in chromosomes 3 and 5 – also presumably due to pericentric inversions. The closely related junco exhibits polymorphisms for pericentric inversions in chromosomes 2 and 5 [65] and the white-crowned sparrow (*Z. leucophrys*) has centric rearrangements in chromosomes 3, 5, and 12 [66]. Thus, chromosomal inversions in *Zonotrichia *and its congeners seem to be relatively common [65,66]. Our comparative analysis of the *VIP *intron [50] revealed that the *Dra*I polymorphism likely existed in the common ancestor of all four North American species of *Zonotrichia*, but not in the South American species, *Z. capensis*. In accordance with these results, sequence data from *VIP *(Figures 9 and 10) and other areas of chromosome 2^m ^suggest that the first inversion predated the divergence of white-throated sparrow from both white-crowned sparrow and Harris's sparrow, originating approximately 2.2 ± 0.3 MYA [62]. Surprisingly, our comparative data on the *VIP *intron suggests that, contrary to Thorneycroft's [31] expectations, chromosome 2^m ^(or at least a portion of it) might actually be "ancestral". We are undertaking further comparative mapping to resolve these alternatives.

In the past few years, genomic resources for avian species have advanced by leaps and bounds, making it now possible to use comparative genomics to determine the genes responsible for differences in white and tan morphology, behavior, and physiology. Chicken chromosome paints revealed that white-throated sparrow chromosome 2 was analogous to chicken chromosome 3 [62], now allowing us to pinpoint candidate genes associated with phenotypic differences. In addition, major advances in the zebra finch genome [67] as well as a white-throated sparrow BAC library (CHORI-264; http://bacpac.chori.org/library.php?id=469), mean that detailed mapping is now possible. Over 1000 microsatellite markers, developed for parentage and population genetics analyses in songbirds [68,69], have been used to construct a predicted passerine genome map based on sequence similarity to the chicken genome. Using microsatellite markers that cross-amplify in the *Zonotrichia *with the detailed pedigrees of over 350 families (data not shown) we will be able to generate linkage maps for this species. Finally, in order to advance conservation and evolutionary genomics in this species, it will be important to identify adaptive genetic variation and the role that the environment has had in selecting for the evolution of alternative genotypes. We have also established a website for the sparrow project: http://www.whitethroatedsparrow.org.

## Conclusion

The retention and capacity for generation of novel adaptive genetic variation in a population is key to conservation efforts. Without maintaining this diversity, given the increased anthropogenic impacts of populations and their habitats, many species will face increased vulnerability to extinction in the near future [70,71]. Conservation genetics has traditionally relied on assays of neutral genetic variation, assuming that it accurately reflects adaptive variation [72]. However, the correlation between variation in adaptive and non-adaptive genetic elements can sometimes be low [72-74], and so a true understanding of the interplay between genes and biodiversity will benefit from the additional data that a broader genomics approach can provide. Here we show the utility of applying such a "genomics approach" to study of two key avian species.

Conservation efforts in the endangered California condor will benefit from the characterization and deeper understanding of the segregating lethal chondrodystrophy mutation. Management of the species' genetic diversity under circumstances of maintaining a viable, self-sustaining, managed population that provides birds for reintroduction, and genetic and demographic augmentation of newly established wild California condor populations in the United States and Mexico is a conservation paradigm that merits the fullest support of scientific prowess in genomics, the health sciences, and behavioral ecology, as well as through public policy, public education and law enforcement. Results gathered from the condor studies will help identify recovery options for other at-risk species as their populations continue to decline and experience genetic bottlenecks.

Genomic studies in the white-throated sparrow are still in their infancy although the potential benefits of this avenue of study are great. Morphs of the white-throated sparrow provide a novel intraspecific comparison of selective pressures, where factors affecting fitness can be linked to specific genes – in other words, we have the opportunity to directly assess adaptive variation. In addition, genomic studies in the sparrow will contribute to our understanding of chromosomal inversions, where suites of genes are often inherited as co-adapted gene complexes and the "success" of a phenotype is highly dependent on orchestrated gene cascades and environmental effects.

Birds are highly influential model organisms in conservation and evolutionary research, and the foundation of work on many avian species is already strong. With the advances of genomic resources for chicken, turkey, zebra finch, and now, the California condor and white-throated sparrow, it is now feasible to expand genomics studies to other avian groups.

## Competing interests

The authors declare that they have no competing interests.

## Authors' contributions

MNR, as a principal researcher for the California condor genome project, performed BAC library screen, PCR amplification and sequencing of the *ACAN *gene and other avian microsatellites, analyzed the results and co-authored the manuscript writing. EMT is principal investigator of the white-throated sparrow project, coordinated the laboratory and field research, and co-authored the manuscript writing. MLH provided karyotyping and G-banding for the white-throated sparrow project, avian cell culture preparation and maintenance. WSM carried out FISH mapping and cell culture preparation for the condor project. LGC was involved in DNA extraction and curation, molecular sexing, and genotype work. MLK assisted in PCR amplification and sequencing for the white-throated sparrow project, and sample collection in the field. EMSM and CAO performed DNA extraction for the condor project and microsatellite genotyping. TR contributed to the re-sequencing of the *ACAN *gene (exon 12), microsatellite genotyping, and molecular sexing. KCJ was responsible for microsatellite library construction supervision, and primer design and validation. SD and JCP directed microsatellite genotyping for the condor project. YD provided resource population construction for the condor project and linkage analysis. EDG and NISC Comparative Sequencing Program supported the sequencing of condor BACs. VM, MTH, JG, SM, ERM carried out 454 sequencing and sequence analyses for the condor project. OAR managed overall condor genome project development and supervision, and contributed to writing the manuscript.

## Supplementary Material

Additional file 1 Pedigree of a condor resource population chosen for microsatellite and linkage analyses, with 121 individuals. Click here for file

Additional file 2 List of overgo probes used for the second screening of the condor BAC library. Click here for file

Additional file 3 **Chicken chromosome map (in Mb) showing location of the 199 loci aligned with the California condor BAC clones (numbers in parentheses)**. Created with MapChart [83] and GenomePixelizer [84] software. With the colored symbols, chicken loci are given that were anchored to condor BACs using California condor (red), New World vulture (gold) and zebra finch (green) sequence derived OVERGOs or Uprobes (blue) followed up by direct sequencing. GGAUn_random, an unknown virtual chicken chromosome sequence generated by the International Chicken Genome Sequencing Consortium [19] that is yet to be assigned to a named chromosome. *LOC770630 *(multiple GGAUn_random?), the probe for this locus may also align with the chicken sequence at additional unresolved locations. *, the *CRES0014 *and *GAPR1 *sequences align with several chicken chromosomes, GGA1 and GGA2 being the best matches, respectively. Click here for file

Additional file 4 Database of condor BACs positive for known avian genes, markers and other sequences Click here for file

Additional file 5 Chicken-condor comparative cytogenetic map showing a considerable degree of conserved synteny.
GGA, *Gallus gallus* (chicken) chromosomes; GCA, *Gymnogyps californianus *(California condor) chromosomes. Click here for file

Additional file 6 A condor-human comparative physical map for HSA7q31 Click here for file

Additional file 7 **Distribution of the condor 454 transcripts homologous to chicken genes**. The histogram illustrates how the condor reads fall into the chicken gene bins where the '0' bin represents all the genes with 0 to 9 members, the '10' bin genes with 10 to 19 reads and so on. There were a lot of rare transcripts (~45,000), with 0 to 20 members, vs. small numbers of other abundant and extremely abundant transcripts, with up to 55,000+ members, in this fibroblast cell line. Click here for file

Additional file 8 **Most abundant transcripts in a transformed condor fibroblast cell line**. Proteins that are expected to be expressed in a fibroblast cell line are highlighted with gray, while one protein (MAD1L1) that could be involved in abnormal cell features and functions is given in bold. Click here for file
