# Supplementary figures and images for: The value of avian genomics to the conservation of wildlife
Source: BMC Genomics. 2009 Jul 14;10(Suppl 2):S10. doi: 10.1186/1471-2164-10-S2-S10 (PMC2966331; doi:10.1186/1471-2164-10-S2-S10)

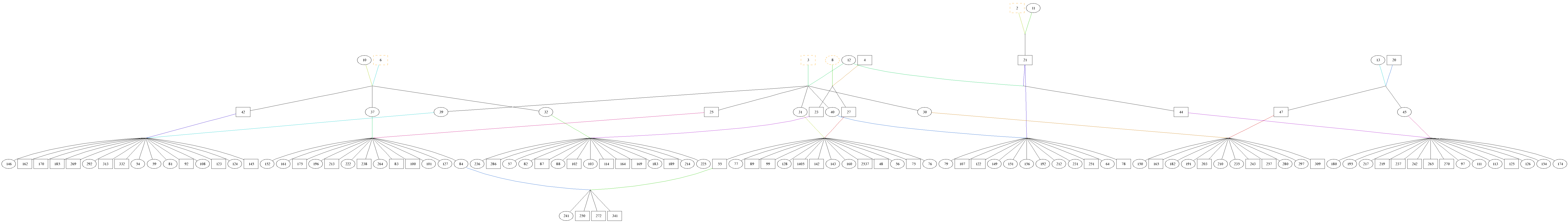

Supplement: Additional file 1 — Pedigree of a condor resource population chosen for microsatellite and linkage analyses, with 121 individuals. [file 1471-2164-10-S2-S10-S1.png]

## Slide 1
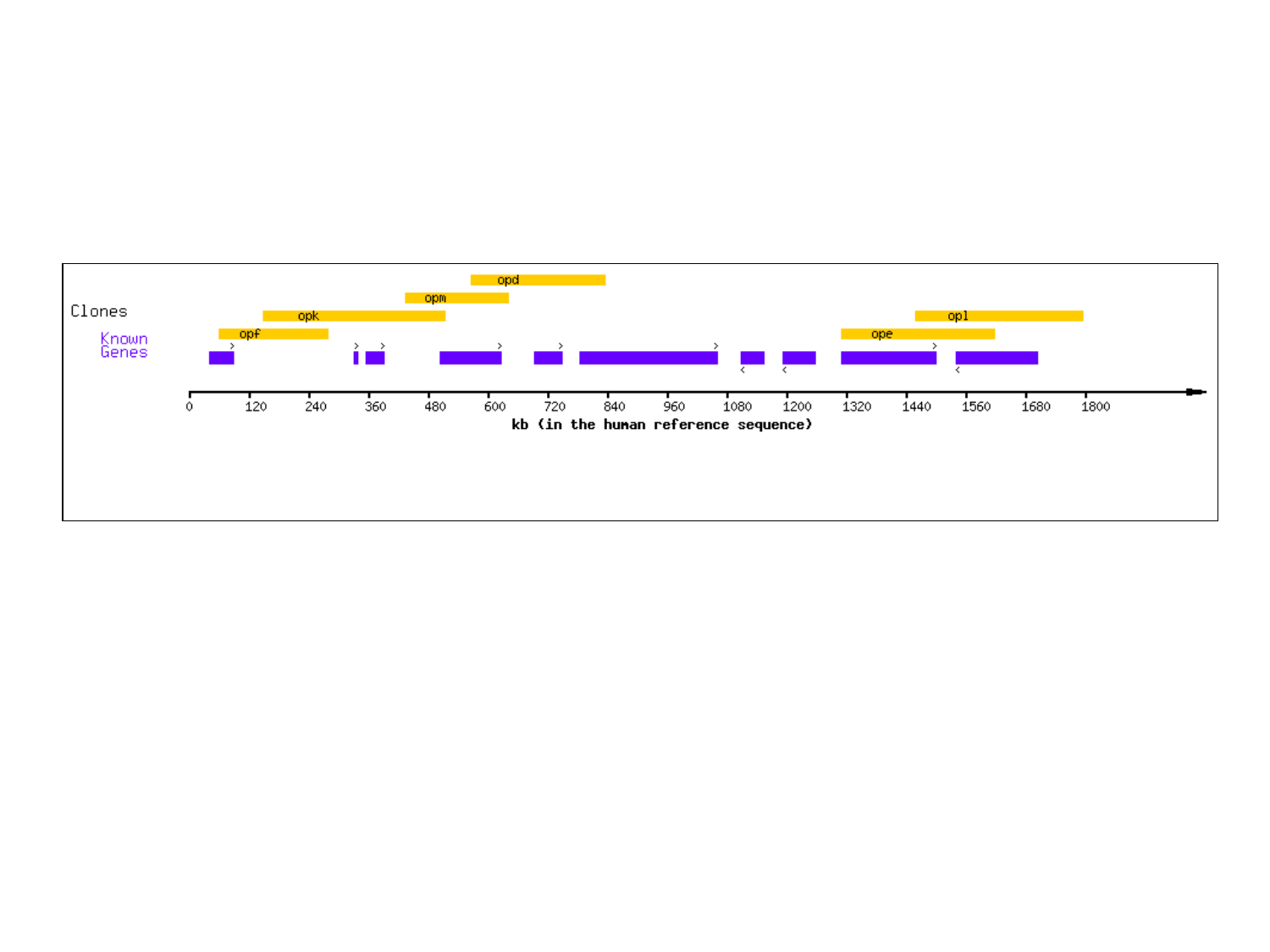

Supplement: Additional file 6 — A condor-human comparative physical map for HSA7q31 [file 1471-2164-10-S2-S10-S6.ppt]

## Slide 1
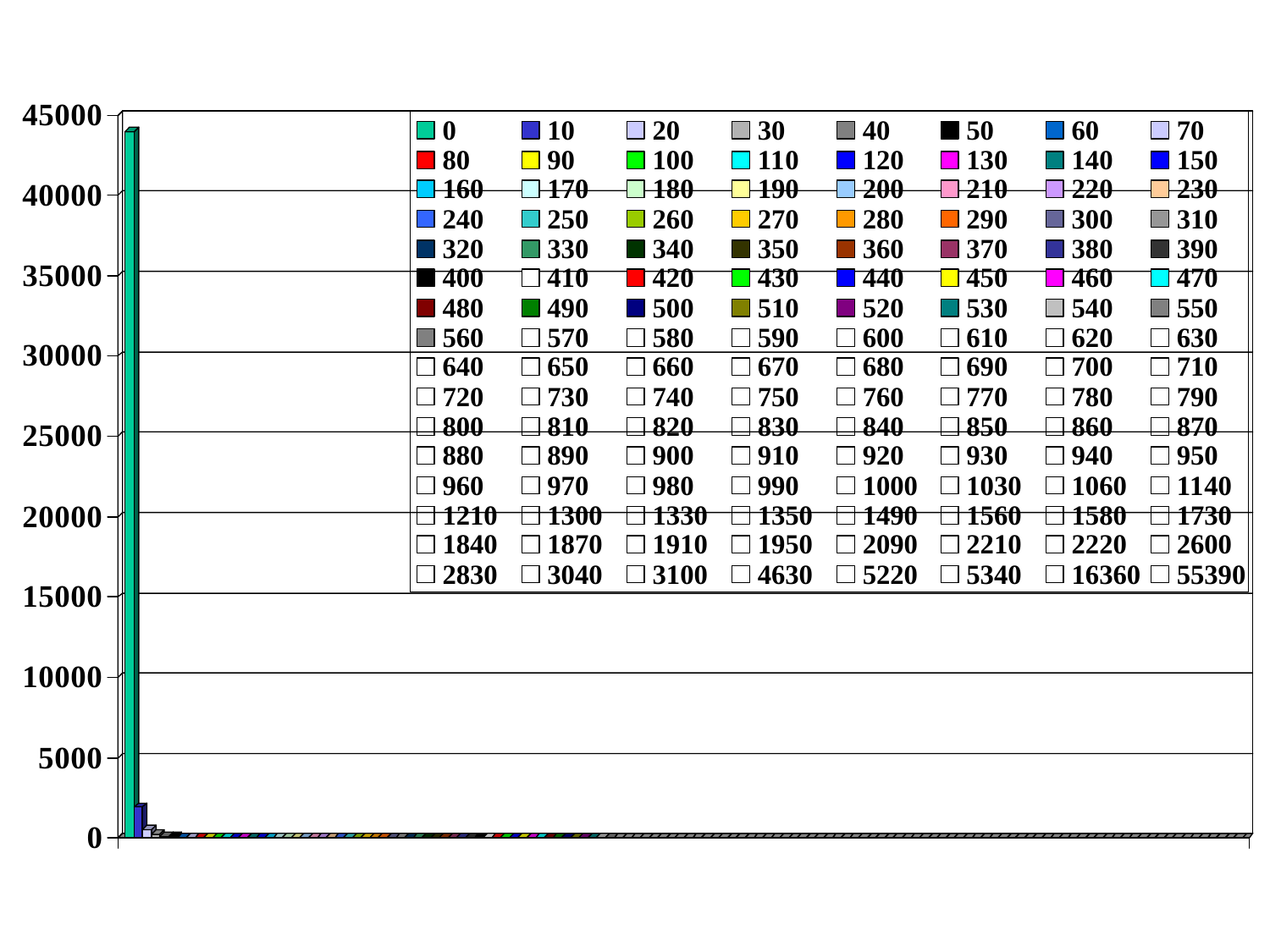

Supplement: Additional file 7 — Distribution of the condor 454 transcripts homologous to chicken genes. The histogram illustrates how the condor reads fall into the chicken gene bins where the '0' bin represents all the genes with 0 to 9 members, the '10' bin genes with 10 to 19 reads and so on. There were a lot of rare transcripts (~45,000), with 0 to 20 members, vs. small numbers of other abundant and extremely abundant transcripts, with up to 55,000+ members, in this fibroblast cell line. [file 1471-2164-10-S2-S10-S7.ppt]
