# Supplementary material for: The value of avian genomics to the conservation of wildlife
Source: BMC Genomics. 2009 Jul 14;10(Suppl 2):S10. doi: 10.1186/1471-2164-10-S2-S10 (PMC2966331; doi:10.1186/1471-2164-10-S2-S10)
Supplement: Additional file 3 — Chicken chromosome map (in Mb) showing location of the 199 loci aligned with the California condor BAC clones (numbers in parentheses). Created with MapChart [83] and GenomePixelizer [84] software. With the colored symbols, chicken loci are given that were anchored to condor BACs using California condor (red), New World vulture (gold) and zebra finch (green) sequence derived OVERGOs or Uprobes (blue) followed up by direct sequencing. GGAUn_random, an unknown virtual chicken chromosome sequence generated by the International Chicken Genome Sequencing Consortium [19] that is yet to be assigned to a named chromosome. LOC770630 (multiple GGAUn_random?), the probe for this locus may also align with the chicken sequence at additional unresolved locations. *, the CRES0014 and GAPR1 sequences align with several chicken chromosomes, GGA1 and GGA2 being the best matches, respectively. [file 1471-2164-10-S2-S10-S3.ppt]

## Slide 1
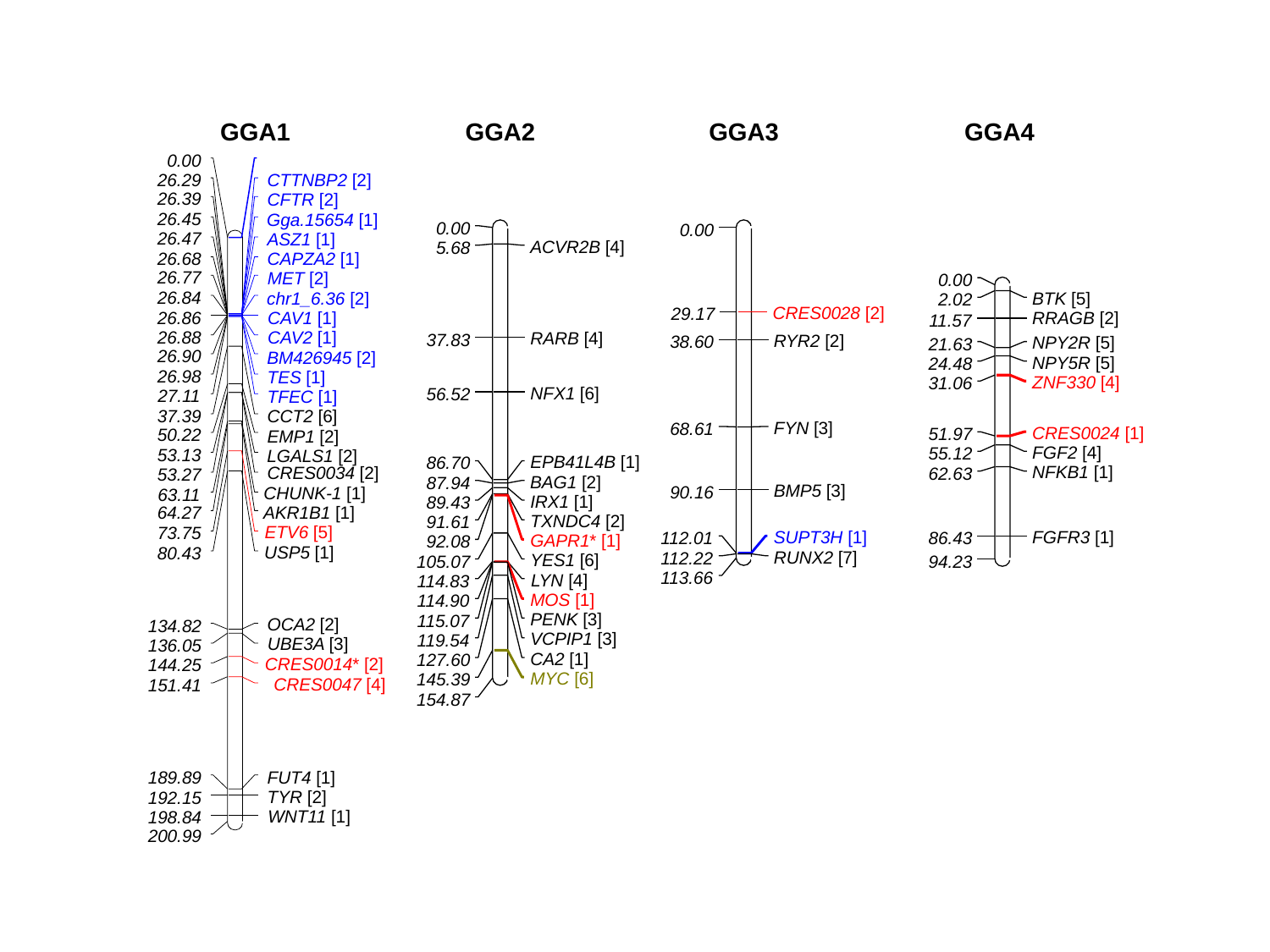

GGA1
GGA2
START
0.00
ACVR2B [4]
5.68
RARB [4]
37.83
NFX1 [6]
56.52
EPB41L4B [1]
86.70
BAG1 [2]
87.94
IRX1 [1]
89.43
TXNDC4 [2]
91.61
GAPR1* [1]
92.08
YES1 [6]
105.07
LYN [4]
114.83
MOS [1]
114.90
PENK [3]
115.07
VCPIP1 [3]
119.54
CA2 [1]
127.60
MYC [6]
145.39
END
154.87
GGA3
GGA4
0.00
START
26.29
CTTNBP2 [2]
26.39
CFTR [2]
26.45
Gga.15654 [1]
START
0.00
26.47
ASZ1 [1]
CAPZA2 [1]
26.68
26.77
MET [2]
START
0.00
26.84
chr1_6.36 [2]
BTK [5]
2.02
CRES0028 [2]
29.17
26.86
RRAGB [2]
CAV1 [1]
11.57
26.88
CAV2 [1]
RYR2 [2]
38.60
NPY2R [5]
21.63
26.90
BM426945 [2]
NPY5R [5]
24.48
26.98
TES [1]
ZNF330 [4]
31.06
27.11
TFEC [1]
37.39
CCT2 [6]
FYN [3]
68.61
CRES0024 [1]
51.97
50.22
EMP1 [2]
FGF2 [4]
55.12
53.13
LGALS1 [2]
NFKB1 [1]
CRES0034 [2]
62.63
53.27
BMP5 [3]
90.16
CHUNK-1 [1]
63.11
64.27
AKR1B1 [1]
ETV6 [5]
73.75
SUPT3H [1]
FGFR3 [1]
112.01
86.43
USP5 [1]
80.43
RUNX2 [7]
112.22
END
94.23
END
113.66
OCA2 [2]
134.82
UBE3A [3]
136.05
CRES0014* [2]
144.25
CRES0047 [4]
151.41
FUT4 [1]
189.89
TYR [2]
192.15
WNT11 [1]
198.84
200.99
END

## Slide 2
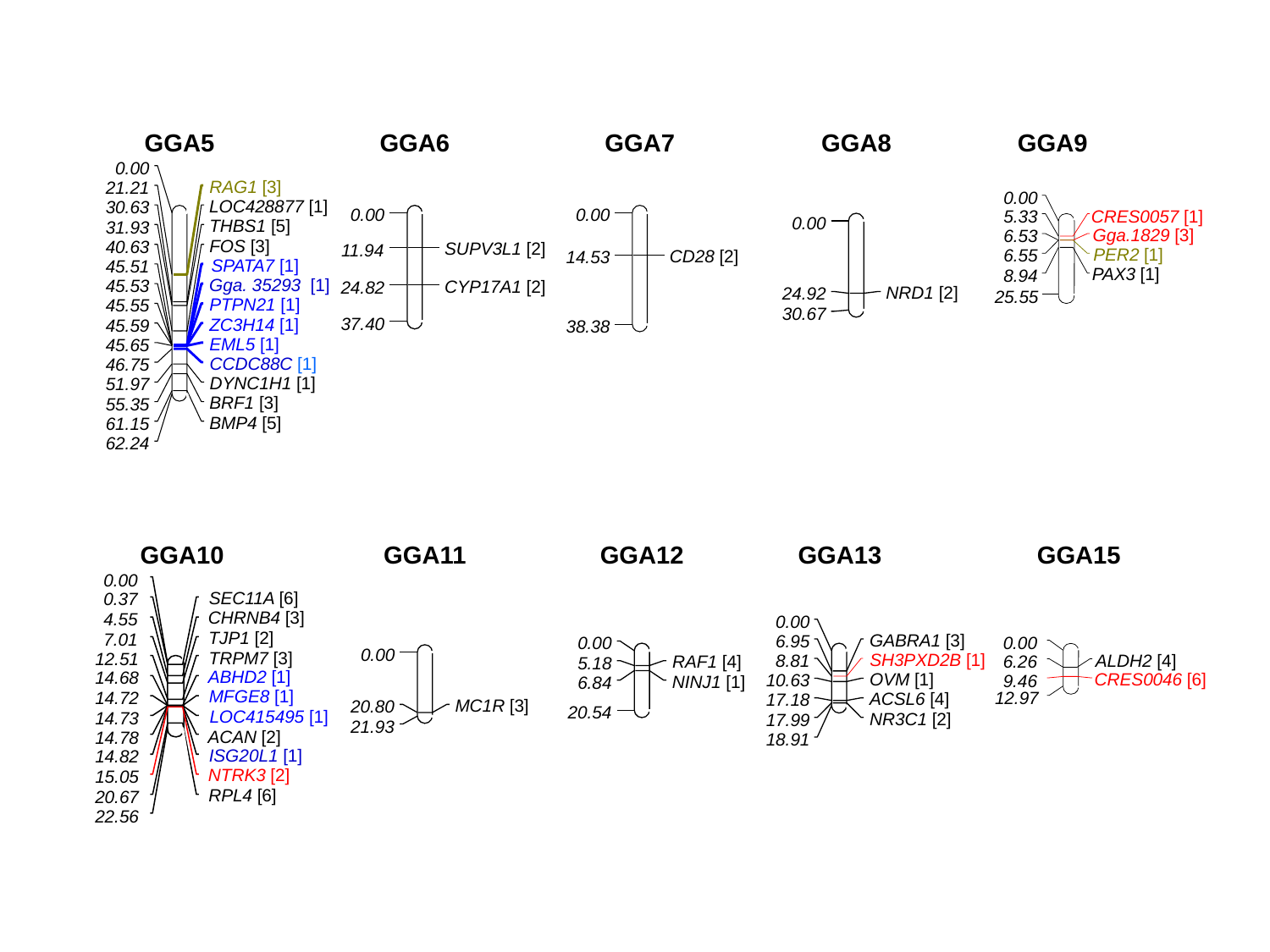

GGA5
GGA6
GGA7
GGA8
GGA9
START
0.00
5.33
CRES0057 [1]
Gga.1829 [3]
6.53
PER2 [1]
6.55
PAX3 [1]
8.94
END
25.55
START
0.00
RAG1 [3]
21.21
LOC428877 [1]
30.63
START
START
0.00
0.00
START
0.00
THBS1 [5]
31.93
FOS [3]
40.63
SUPV3L1 [2]
11.94
CD28 [2]
14.53
SPATA7 [1]
45.51
Gga. 35293 [1]
45.53
CYP17A1 [2]
24.82
NRD1 [2]
24.92
PTPN21 [1]
45.55
END
30.67
END
37.40
ZC3H14 [1]
45.59
END
38.38
EML5 [1]
45.65
CCDC88C [1]
46.75
DYNC1H1 [1]
51.97
BRF1 [3]
55.35
BMP4 [5]
61.15
END
62.24
GGA10
GGA11
GGA12
GGA13
GGA15
START
0.00
SEC11A [6]
0.37
CHRNB4 [3]
4.55
START
0.00
TJP1 [2]
7.01
GABRA1 [3]
6.95
START
START
0.00
0.00
START
0.00
MC1R [3]
20.80
END
21.93
TRPM7 [3]
12.51
SH3PXD2B [1]
ALDH2 [4]
8.81
6.26
RAF1 [4]
5.18
ABHD2 [1]
14.68
OVM [1]
CRES0046 [6]
10.63
9.46
NINJ1 [1]
6.84
MFGE8 [1]
14.72
12.97
END
ACSL6 [4]
17.18
END
20.54
LOC415495 [1]
14.73
NR3C1 [2]
17.99
ACAN [2]
14.78
END
18.91
ISG20L1 [1]
14.82
NTRK3 [2]
15.05
RPL4 [6]
20.67
END
22.56

## Slide 3
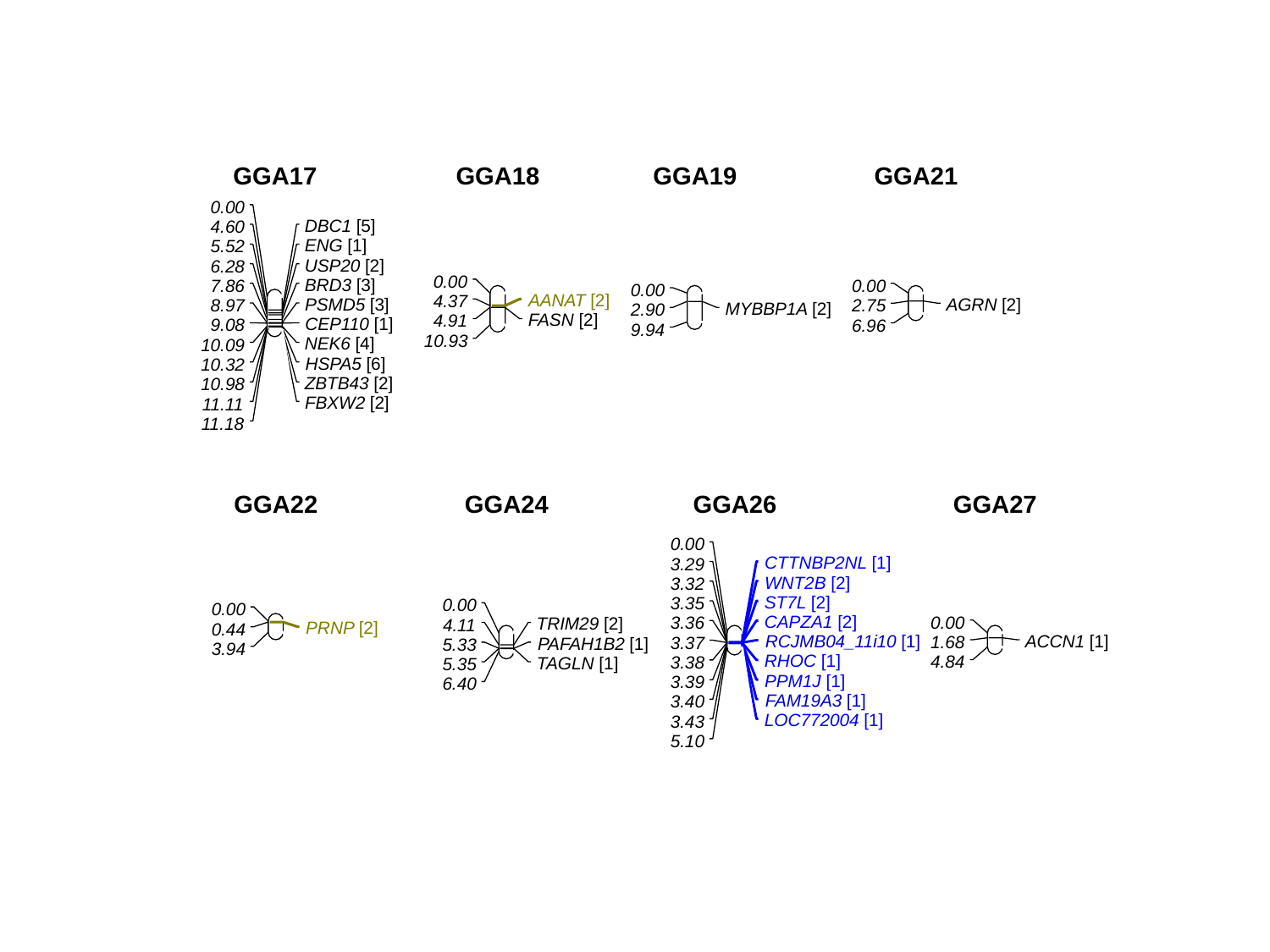

GGA17
START
0.00
DBC1 [5]
4.60
ENG [1]
5.52
USP20 [2]
6.28
BRD3 [3]
7.86
PSMD5 [3]
8.97
CEP110 [1]
9.08
NEK6 [4]
10.09
HSPA5 [6]
10.32
ZBTB43 [2]
10.98
FBXW2 [2]
11.11
END
11.18
GGA18
GGA19
GGA21
START
0.00
START
0.00
START
0.00
AANAT [2]
4.37
AGRN [2]
2.75
MYBBP1A [2]
2.90
FASN [2]
4.91
END
6.96
END
9.94
END
10.93
GGA22
START
0.00
PRNP [2]
0.44
END
3.94
GGA24
GGA26
GGA27
START
0.00
CTTNBP2NL [1]
3.29
WNT2B [2]
3.32
ST7L [2]
3.35
START
0.00
START
CAPZA1 [2]
0.00
3.36
TRIM29 [2]
4.11
ACCN1 [1]
RCJMB04_11i10 [1]
1.68
3.37
PAFAH1B2 [1]
5.33
END
RHOC [1]
4.84
3.38
TAGLN [1]
5.35
PPM1J [1]
3.39
END
6.40
FAM19A3 [1]
3.40
LOC772004 [1]
3.43
END
5.10

## Slide 4
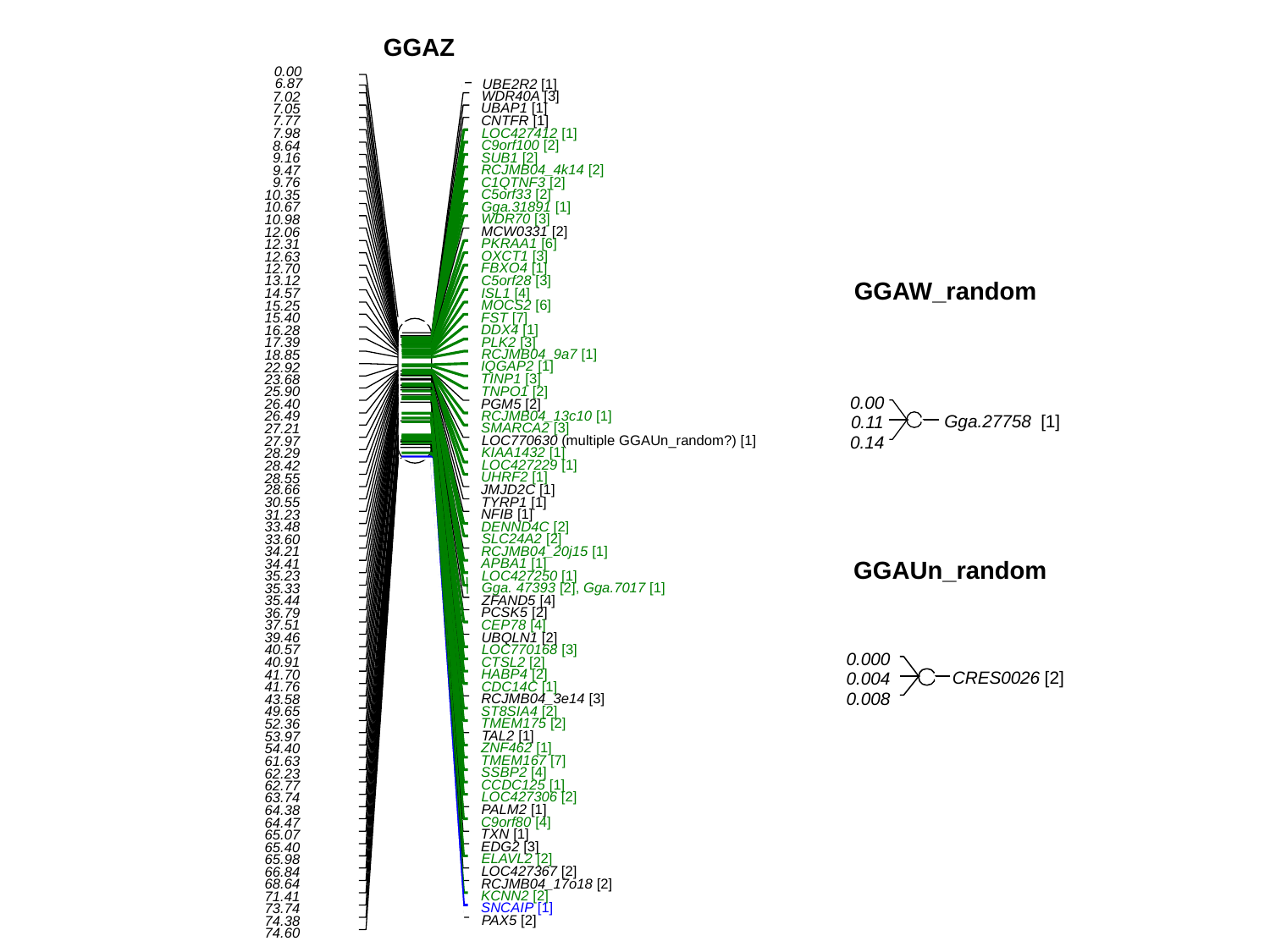

GGAZ
0.00
6.87
START
UBE2R2 [1]
WDR40A [3]
7.02
UBAP1 [1]
7.05
CNTFR [1]
7.77
LOC427412 [1]
7.98
C9orf100 [2]
8.64
SUB1 [2]
9.16
RCJMB04_4k14 [2]
9.47
C1QTNF3 [2]
9.76
C5orf33 [2]
10.35
Gga.31891 [1]
10.67
WDR70 [3]
10.98
MCW0331 [2]
12.06
PKRAA1 [6]
12.31
OXCT1 [3]
12.63
FBXO4 [1]
12.70
C5orf28 [3]
13.12
ISL1 [4]
14.57
MOCS2 [6]
15.25
FST [7]
15.40
DDX4 [1]
16.28
PLK2 [3]
17.39
RCJMB04_9a7 [1]
18.85
IQGAP2 [1]
22.92
TINP1 [3]
23.68
TNPO1 [2]
25.90
PGM5 [2]
26.40
RCJMB04_13c10 [1]
26.49
SMARCA2 [3]
27.21
LOC770630 (multiple GGAUn_random?) [1]
27.97
KIAA1432 [1]
28.29
LOC427229 [1]
28.42
UHRF2 [1]
28.55
28.66
JMJD2C [1]
30.55
TYRP1 [1]
NFIB [1]
31.23
DENND4C [2]
33.48
SLC24A2 [2]
33.60
RCJMB04_20j15 [1]
34.21
APBA1 [1]
34.41
LOC427250 [1]
35.23
Gga. 47393 [2], Gga.7017 [1]
35.33
ZFAND5 [4]
35.44
PCSK5 [2]
36.79
CEP78 [4]
37.51
UBQLN1 [2]
39.46
LOC770168 [3]
40.57
CTSL2 [2]
40.91
HABP4 [2]
41.70
CDC14C [1]
41.76
RCJMB04_3e14 [3]
43.58
ST8SIA4 [2]
49.65
TMEM175 [2]
52.36
TAL2 [1]
53.97
ZNF462 [1]
54.40
TMEM167 [7]
61.63
SSBP2 [4]
62.23
CCDC125 [1]
62.77
LOC427306 [2]
63.74
PALM2 [1]
64.38
C9orf80 [4]
64.47
TXN [1]
65.07
EDG2 [3]
65.40
ELAVL2 [2]
65.98
LOC427367 [2]
66.84
RCJMB04_17o18 [2]
68.64
KCNN2 [2]
71.41
SNCAIP [1]
73.74
PAX5 [2]
74.38
END
74.60
GGAW_random
START
0.00
Gga.27758 [1]
0.11
END
0.14
GGAUn_random
START
0.000
CRES0026 [2]
0.004
END
0.008
