# Supplementary material for: The value of avian genomics to the conservation of wildlife
Source: BMC Genomics. 2009 Jul 14;10(Suppl 2):S10. doi: 10.1186/1471-2164-10-S2-S10 (PMC2966331; doi:10.1186/1471-2164-10-S2-S10)
Supplement: Additional file 5 — Chicken-condor comparative cytogenetic map showing a considerable degree of conserved synteny. GGA, Gallus gallus (chicken) chromosomes; GCA, Gymnogyps californianus (California condor) chromosomes. [file 1471-2164-10-S2-S10-S5.ppt]

## Slide 1
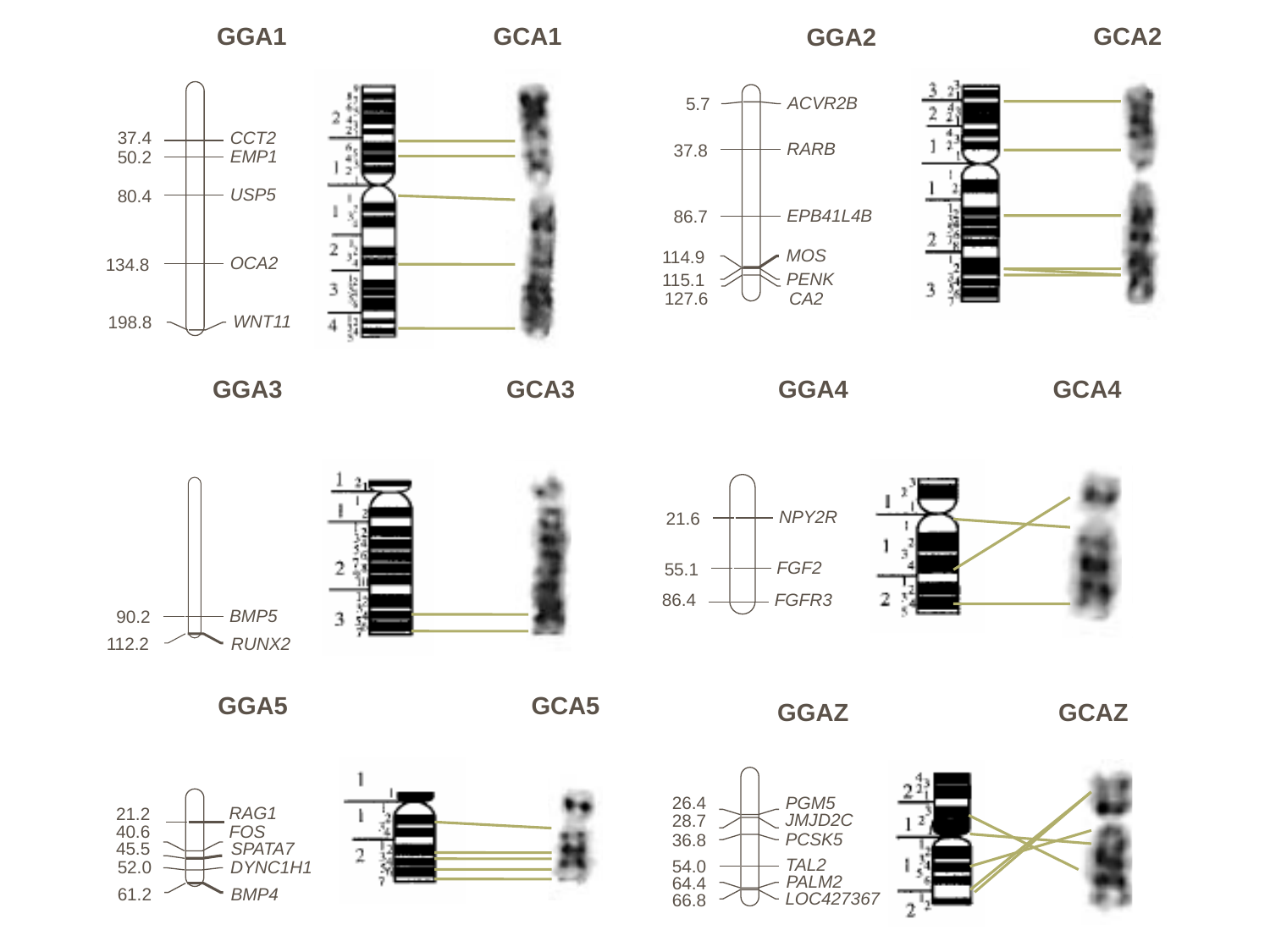

GGA1
GCA1
37.4
CCT2
EMP1
50.2
USP5
80.4
OCA2
134.8
WNT11
198.8
GCA2
GGA2
ACVR2B
5.7
RARB
37.8
EPB41L4B
86.7
MOS
114.9
PENK
115.1
127.6
CA2
GGA3
GCA3
BMP5
90.2
112.2
RUNX2
GGA4
GCA4
NPY2R
21.6
FGF2
55.1
86.4
FGFR3
GGA5
GCA5
RAG1
21.2
40.6
FOS
45.5
SPATA7
52.0
DYNC1H1
61.2
BMP4
GGAZ
GCAZ
26.4
PGM5
JMJD2C
28.7
PCSK5
36.8
TAL2
54.0
PALM2
64.4
LOC427367
66.8
