# Supplementary material for: The value of avian genomics to the conservation of wildlife
Source: BMC Genomics. 2009 Jul 14;10(Suppl 2):S10. doi: 10.1186/1471-2164-10-S2-S10 (PMC2966331; doi:10.1186/1471-2164-10-S2-S10)
Supplement: Additional file 8 — Most abundant transcripts in a transformed condor fibroblast cell line. Proteins that are expected to be expressed in a fibroblast cell line are highlighted with gray, while one protein (MAD1L1) that could be involved in abnormal cell features and functions is given in bold. [file 1471-2164-10-S2-S10-S8.doc]

| **No. of reads per gene** | **P-value** | **Human homolog** |
| --- | --- | --- |
| **55391** | **7.7e-104** | **NM_003550.2| MAD1 mitotic arrest deficient-like 1(yeast) (MAD1L1)** |
| 16363 | 9.0e-14 | NM_005807.2| proteoglycan 4 (PRG4), mRNA |
| 5349 | 0.0017 | NM_001012709.1| keratin associated protein 5-4 (KRTAP5-4) |
| 5221 | 5.6e-128 | NM_002345.3| lumican (LUM) |
| 4637 | 6.0e-05 | NM_005807.2| proteoglycan 4 (PRG4) |
| 3107 | 0.18 | NM_138300.3| pygopus homolog 2 (PYGO2) |
| 3047 | 4.8e-147 | NM_000133.2| coagulation factor IX (plasma thromboplastic component, Christmas disease, hemophilia B) (F9) |
| 2836 | 5.1e-85 | NM_021009.3| ubiquitin C (UBC) |
| 2608 | 0.19 | NM_004409.2| dystrophia myotonica-protein kinase (DMPK) |
| 2220 | 0.015 | NM_198572.1| spermatogenesis and centriole associated 1 (SPATC1) |
| 2215 | 2.8e-157 | NM_005566.1| lactate dehydrogenaseA (LDHA) |
| 2097 | 0. | NM_006597.3| heat shock 70kDa protein 8 (HSPA8), transcript variant 1 |
| 1957 | 3.4e-131 | NM_001920.3| decorin (DCN) |
| 1918 | 5.5e-102 | NM_007155.4| zona pellucida glycoprotein 3 (sperm receptor) (ZP3) |
| 1871 | 6.4e-156 | NM_021009.3| ubiquitin C (UBC) |
| 1846 | 9.7e-66 | NM_001004724.1| olfactory receptor, family 4, subfamily N, member 5 (OR4N5) |
| 1735 | 1.1e-176 | NM_000604.2| fibroblast growth factor receptor 1 (fms-related tyrosine kinase 2, Pfeiffer syndrome) (FGFR1) |
| 1588 | 1.5e-203 | NM_001613.1| actin, alpha 2, smooth muscle, aorta (ACTA2) |
| 1563 | 1.9e-49 | NM_000567.2| C-reactive protein, pentraxin-related (CRP) |
| 1491 | 7.9e-226 | NM_005348.2| heat shock protein 90kDa alpha (cytosolic), class A member 1 (HSP90AA1) |
